# Autophagy, a novel player in acetaminophen (APAP)-induced hepatotoxicity

**DOI:** 10.1080/27694127.2022.2103779

**Published:** 2022-07-29

**Authors:** Hui Ye, Francisco Javier Cubero

**Affiliations:** a Department of Anesthesiology, ZhongDa Hospital Southeast University, 210009 Nanjing, China; b Department of Immunology, Ophthalmology and ENT, Complutense University School of Medicine, 28040 Madrid, Spain; c Centro de Investigación Biomédica en Red de Enfermedades Hepáticas y Digestivas (CIBEREHD), 28029 Madrid, Spain; d Instituto de Investigación Sanitaria Gregorio Marañón (IiSGM), 28007 Madrid, Spain

**Keywords:** Acetaminophen (APAP), autophagy, endoplasmic reticulum (ER) stress, ERN1, liver

## Abstract

Acetaminophen (APAP), a widely used analgesic drug, is safe at therapeutic doses, but can produce significant hepatotoxicity upon overdose. Emerging evidence suggested that endoplasmic reticulum (ER) stress and the unfolded protein response (UPR) are key mechanisms in APAP-mediated hepatotoxicity. While ER stress-UPR and macroautophagy/autophagy appear to be independent cellular processes, we found a cross-linked mechanism in APAP liver injury. The specific ablation in liver parenchyma of XBP1 (X-box binding protein 1), a transcription factor mediating ER stress, mitigates APAP-induced liver injury. Interestingly, this mechanism is linked to enhanced autophagy which seems to be responsible for the modulation of the enzymatic activity of CYP2E1, which is involved in the metabolic conversion of APAP, and ultimately protects liver against APAP toxicity. Altogether, our study highlighted autophagy as a novel player in the pathophysiology of APAP-induced liver injury and opened new therapeutic avenues for its modulation in patients with drug overdose.

Acetaminophen (APAP) overdose has been the most frequent cause of acute liver failure (ALF) and severe liver injury in western countries and the second most common cause of liver transplantation worldwide. Approximately 10% of APAP is metabolized by CYP2E1 (cytochrome P450 family 2 subfamily E member 1) enzymes, to form a reactive metabolite, *N*-acetyl-*p*-benzoquinone imine (NAPQI), which at a toxic dose depletes hepatic glutathione stores, resulting in mitochondrial dysfunction, oxidative stress and, more importantly, in endoplasmic reticulum (ER) stress. Perturbations in ER function trigger the unfolded protein response (UPR), a pro-survival process; however, sustained and/or prolonged stress may result in cell death induction. Here, the mode of hepatocyte death is still a matter of debate. Both experimental and human data do not support the relevance for apoptosis in APAP-induced liver injury. Conversely, compelling evidence suggests a pivotal role for necrosis in APAP-induced hepatotoxicity, whereas the relevance of necroptosis remains to be further elucidated.

Emerging evidence indicates that autophagy plays a critical role in protecting against APAP-induced liver injury. Autophagy is a catabolic process that degrades and recycles excess or aberrant organelles and misfolded proteins through the lysosomal machinery. Thus, in our published study, we explored the role of APAP-induced hepatic ER stress. Specifically, we focused on the activation of the ERN1/IRE1-XBP1 pathway, the most conserved branch of the UPR, in which spliced XBP1 (XBP1s) is a transcription factor that induces genes involved in chaperoning proteins through the ER and degrading proteins that cannot be properly folded. Interestingly, we unveiled a novel mechanism of protection against APAP by which decreased XBP1s induces autophagy.

Our data [1] clearly show that XBP1 splicing is involved in human and murine APAP-induced pathophysiology. Therefore, we generated mice with specific deletion of *Xbp1* in hepatocytes (*xbp1^Δhepa^*). *xbp1* deletion causes ERN1 overactivation through a feedback-modulated mechanism, and the resulting activation of regulated ERN1-depedent decay/RIDD leading to the suppression of CYP2E1 enzyme activity (Figure 1). This explains why CYP2E1 levels are evidently decreased both at early and late phases of APAP hepatotoxicity in *Xbp1*-deficient animals. Consequently, restoration of the GSH system reduces the generation of reactive oxygen species/ROS and prevents severe oxidative stress and hepatocellular injury. Interestingly, the cytoprotective effect after high-dose APAP exposure was confirmed by reduced DDIT3/CHOP, HSPA5/BiP/GRP78 and MAPK/JNK activation in *xbp1^Δhepa^* animals (Figure 1).

**Figure 1.**
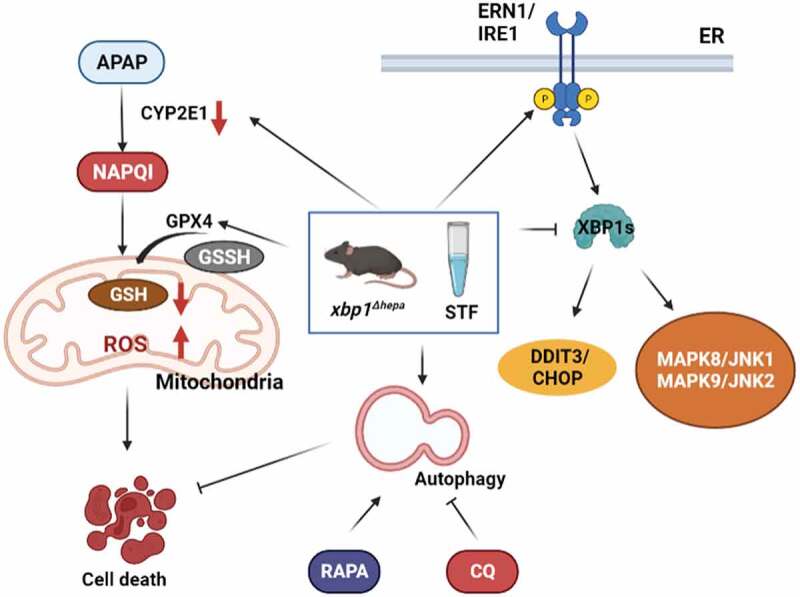
Schematic representation of the interplay between ER stress-UPR and autophagy during APAP-mediated liver injury. APAP is converted into NAPQI by the action of CYP2E1, generating reactive oxygen species (ROS) and glutathione (GSH) depletion and activation of ER stress (XBP1s-DDIT3/CHOP) and MAPK/JNK translocation to the mitochondria, triggering cell death. Specific deletion of *Xbp1* in hepatocytes (*xbp1^∆hepa^*) and/or STF pretreatment enhance APAP-mediated hepatotoxicity via activation of autophagy, as validated by modulation of autophagy with rapamycin (RAPA) or chloroquine (CQ) treatment.

However, the protective mechanism was still to be understood. It is well-know that autophagosomes are the morphological hallmark of autophagy and the number of autophagosomes is an indicator of the level of autophagic activity. Therefore, we checked our APAP-treated mice using transmission electron microscopy (TEM) and found abundant autophagosome formation in *xbp1^Δhepa^* mice upon APAP toxicity. This gave us the hint that autophagy could be responsible for the cytoprotective mechanism observed in *Xbp1*-deficient animals.

Next, the molecular mechanisms related to autophagy were investigated. Hepatocytic *Xbp1* deficiency significantly promotes the phosphorylation of AMPK, the expression of *Atg5*, the conversion of MAP1LC3/LC3 (microtubule-associated protein 1 light chain 3)-I to LC3-II and the degradation of SQSTM1/p62 following APAP intoxication, linking *xbp1* deletion to autophagy both at early and late stages of APAP-induced liver injury. Moreover, pharmacological use of XBP1 inhibition was tested with STF-083010, an inhibitor of the endonuclease activity of ERN1, which markedly mitigates oxidative burst and promotes autophagy activation, after APAP challenge.

To understand the relevance of autophagy during murine APAP-mediated liver injury, we subsequently tested autophagy induction and blockade using rapamycin and chloroquine, respectively, before and after APAP-induced hepatotoxicity. Pretreatment with rapamycin or chloroquine revert the decreased CYP2E1 levels in *Xbp1^f/f^* mice after APAP exposure but have no effect on decreased basal CYP2E1 levels in *xbp1^Δhepa^* livers, indicating that autophagy might be responsible for reduced CYP2E1 enzyme activity in *xbp1^Δhepa^* mice. Moreover, induction of autophagy works even at late stages of APAP metabolism when hepatic GSH depletion has already occurred.

Thus, autophagy is strongly activated in APAP-induced hepatotoxicity to eliminate damaged mitochondria and maintain mitochondrial homeostasis, which is the major source of intracellular oxidative stress. Our data indicate that promotion of autophagy might be a plausible therapeutic strategy to prevent and treat APAP-induced liver injury. As an important mechanism for maintaining cellular homeostasis, autophagy can clear APAP protein adducts and eliminate damaged mitochondria. At present, the molecular mechanisms that link the UPR to autophagy remain poorly understood. Our results open a new line of research which merits exploration in order to find novel pharmacological treatments for patients with toxin-induced liver injury. The induction of autophagy, linked to the decreased expression of XBP1, may have a potential therapeutic application in patients suffering from APAP intoxication. In summary, this study provides new mechanistic information on the role of ER stress-UPR and brings a novel player – autophagy – into APAP-mediated hepatotoxicity.
